# Novel Therapy for Glioblastoma Multiforme by Restoring LRRC4 in Tumor Cells: LRRC4 Inhibits Tumor-Infitrating Regulatory T Cells by Cytokine and Programmed Cell Death 1-Containing Exosomes

**DOI:** 10.3389/fimmu.2017.01748

**Published:** 2017-12-11

**Authors:** Peiyao Li, Jianbo Feng, Yang Liu, Qiang Liu, Li Fan, Qing Liu, Xiaoling She, Changhong Liu, Tao Liu, Chunhua Zhao, Wei Wang, Guiyuan Li, Minghua Wu

**Affiliations:** ^1^Hunan Provincial Tumor Hospital and the Affiliated Tumor Hospital of Xiangya Medical School, Central South University, Changsha, China; ^2^Key Laboratory of Carcinogenesis and Cancer Invasion, Ministry of Education, Key Laboratory of Carcinogenesis, Ministry of Health, Cancer Research Institute, Central South University, Changsha, China; ^3^Xiangya Hospital, Central South University, Changsha, China; ^4^Third Xiangya Hospital, Central South University, Changsha, China; ^5^Department of Biochemistry, University of California, Riverside, CA, United States; ^6^Second Xiangya Hospital, Central South University, Changsha, China

**Keywords:** CCR4, exosome, programmed cell death 1, regulatory T cells, tumor-infiltrating lymphocytes

## Abstract

Glioblastoma multiforme (GBM) is a heterogeneous malignant brain tumor, the pathological incidence of which induces the accumulation of tumor-infiltrating lymphocytes (TILs). As a tumor suppressor gene, LRRC4 is absent in GBM cells. Here, we report that the recovery of LRRC4 in GBM cells inhibited the infiltration of tumor-infiltrating regulatory T cells (Ti-Treg), promoted the expansion of tumor-infiltrating effector T (Ti-Teff) cells and CD4^+^CCR4^+^ T cells, and enhanced the chemotaxis of CD4^+^CCR4^+^ T cells in the GBM immune microenvironment. LRRC4 was not transferred into TILs from GBM cells through exosomes but mainly exerted its inhibiting function on Ti-Treg cell expansion by directly promoting cytokine secretion. GBM cell-derived exosomes (cytokine-free and programmed cell death 1 containing) also contributed to the modulation of LRRC4 on Ti-Treg, Ti-Teff, and CD4^+^CCR4^+^ T cells. In GBM cells, LRRC4 directly bound to phosphoinositide-dependent protein kinase 1 (PDPK1), phosphorylated IKKβser181, facilitated NF-κB activation, and promoted the secretion of interleukin-6 (IL-6), CCL2, and interferon gamma. In addition, HSP90 was required to maintain the interaction between LRRC4 and PDPK1. However, the inhibition of Ti-Treg cell expansion and promotion of CD4^+^CCR4^+^ T cell chemotaxis by LRRC4 could be blocked by anti-IL-6 antibody or anti-CCL2 antibody, respectively. miR-101 is a suppressor gene in GBM. Our previous studies have shown that EZH2, EED, and DNMT3A are direct targets of miR-101. Here, we showed that miR-101 reversed the hypermethylation of the LRRC4 promoter and induced the re-expression of LRRC4 in GBM cells by directly targeting EZH2, EED, and DNMT3A. Our results reveal a novel mechanism underlying GBM microenvironment and provide a new therapeutic strategy using re-expression of LRRC4 in GBM cells to create a permissive intratumoral environment.

## Introduction

Glioblastoma multiforme (GBM) is the most common malignant brain tumor and has a median survival of only 14.6 months even after treatment. The 2016 central nervous system (CNS) world health organization presents a restructuring of GBM and incorporates new entities that are defined by genetic characteristics and histology, including glioblastoma, IDH-mutant; glioblastoma, NOS, and glioblastoma, IDH-wild type. In addition to IDH, other molecular features (i.e., TP53 mutation and 1p/19q deletion status) are also used to narrowly define entities leading to better diagnostic accuracy and more accurate determinations of treatment response and prognosis (1). GBM has a heterogeneous composition of true tumor cells and range of intermingling non-tumor cells, which also have vital roles in controlling the course of the pathology. In fact, the pathological incidence of a brain tumor induces the accumulation of tumor-specific immune cells, which constitute a part of the brain tumor mass. These are composed of microglia, the resident immune cells of the CNS, and tumor-infiltrating lymphocytes (TILs) derived from outside the CNS, as well as even CNS lymphatic vessels. TILs include CD4^+^CD25^+^FoxP3^+^ regulatory T (Treg) cells, effector T (Teff) cells, and cytotoxic T cells, among others (2–5). Each of these TIL subsets exerts different functions and secretes specific cytokines that can affect tumor growth. Tumor-infiltrating effector Th1 cells can exert direct antitumor cytolysis and secrete IFNγ, which sustains innate cell activation against cancer. Th1 cells also secrete multifunctional cytokines TNFα and IL-2. The latter promotes tumor-infiltrating cytotoxic CD8 T cell differentiation and the release of perforin, granzyme B, IFNγ, and TNFα, which drive tumor regression. Tumor-infiltrating effector Th17 cells can support antitumor responses and augment effector Th and cytotoxic T cell activity. Tumor-infiltrating Treg cells expressing FoxP3 produce IL-10 and exert antitumor effects by inhibiting tumor-infiltrating effector T cells in the tumor microenvironment (6–10). The GBM microenvironment is characterized by high levels of many cytokines. CCL2 secreted by GBM cells binds to its receptor on CCR4^+^ cells to cause them to migrate to the tumor microenvironment and surround the GBM (Ti-CCR4 cells). CCR4 is expressed on most Treg cells, and the binding of CCR4 and CCL2 has been shown to induce Treg cell infiltration across the blood–brain barrier into the parenchyma (Ti-Treg cells). The CCL2–CCR4 axis is required for Treg cell recruitment, and CCR4^−^ deficient Treg cells show defective recruitment in brain tumors. CCL2 consistently exerts a dual role in the tumor microenvironment. CCR4^+^ is also expressed on Teff (Th2 and Th17, among others) cells, and CCL2 mediates CCR4^+^ Teff cell infiltration of the GBM microenvironment (11–13). GBM progression is enabled by immunosuppression driven by immunosuppressive cells involving Ti-Treg cells, whereas Ti-Teff cells possess tumor-suppressing capabilities (3, 14). In addition to Ti-Treg cells, there are multiple, redundant immunosuppressive mechanisms associated with GBM, such as immunosuppressive cytokines (TGF-β, PGE2, among others), immune checkpoints [PDL-1, programmed cell death 1 (PD-1), and CTLA-4] and others (3, 15–17). A detailed understanding of communication between GBM cells and tumor-specific TILs in the GBM microenvironment will help to unveil the key regulatory hub of immunosuppressive mechanisms and contribute to GBM immunotherapy.

Leucine-rich repeat (LRR) C4 protein (LRRC4, netrin-G1 ligand-2) is a new partner of PAR6, PAR3, and PKCζ complex in axon differentiation. LRRC4 regulates the formation of excitatory synapses and promotes neurite outgrowth (18, 19). Previous studies have confirmed that LRRC4 is a tumor suppressor gene for glioma, is capable of regulating miR-182 and miR-381 and constitutes multiphase circuits with transcription factors, gene methylation modifications and miRNAs in GBMs, such as the LRRC4/NGL-2-miR-185/SP1-DNMT1-LRRC4/NGL-2 loop and the LRRC4/NGL-2-AP-2-miR-182-LRRC4/NGL-2 loop. The re-introduction of LRRC4 inhibits GBM cell proliferation, migration, and angiogenesis by downregulating pleiotropic cytokines (IGF, EGF, VEGF, SDF-1α, among others) (20–23).

LRRC4 is a member of the LRR superfamily. The LRR domain is present in numerous proteins. In humans, 375 LRRC proteins have been characterized in innate immunity (24, 25). LRRC4 is decreased in WHO grades II and III astrocytomas and is consistently absent in GBM (20). Conversely, the Treg cell fraction of TILs in tumors increases with the astrocytomas grade, and GBM is characterized by the greatest accumulation of Treg cells. Infiltration of Treg cells promotes the development and progression of GBM (4, 26, 27). These findings suggest a role for LRRC4 in the GBM microenvironment, and we speculate that the absence of LRRC4 may contribute to the infiltration of Treg cells in GBM.

Our previous study has demonstrated a lower expression of miR-101 in glioma tissues than in normal brain tissues. Reduced miR-101 expression is frequently observed in human glioma tissues, and the reduction of miR-101 expression is not correlated with the tumor grade. miR-101 suppresses the expression of EZH2, EED, and DNMT3A, inhibits the activity of H3K4 demethylase, H3K27 methyltransferase, and DNMT3A methyltransferase, and increases the activity of H3K9 and DNMT methyltransferase. In addition, miR-101 indirectly suppresses the expression of CPEB1, ELFN2, and PRDM16 and affects their methylation levels by targeting EZH2, EED and DNMT3A in glioma cells. The methylation-mediated LRRC4 inactivation is a frequent event in astrocytoma, and we found that the LRRC4 promoter was methylated in all glioma cell lines and primary gliomas but not in normal brain tissue. The DNA demethylating agent, 5-Aza-2′-deoxycytidine (5-Aza-dC) reversed LRRC4 hypermethylation and induced LRRC4 re-expression in GBM cells (20, 28). Thus, we propose that reversing the hypermethylation of the LRRC4 promoter to restore LRRC4 expression may contribute to the treatment of GBM.

Here, we focused on the role of LRRC4 in the GBM immune microenvironment by examining tumor tissues, patient peripheral blood and primary GBM cells. Our results showed the following. 1. LRRC4 inhibited the infiltration of Ti-Treg cells, promoted the expansion of Ti-Teff cells and CD4^+^CCR4^+^ T cells, and enhanced the chemotaxis of CD4^+^CCR4^+^ T cells in the GBM microenvironment by promoting cytokine secretion and affecting GBM cell-derived exosomes (cytokine-free and PD-1-containing). 2. LRRC4 bound to phosphoinositide dependent protein kinase 1 (PDPK1) and HSP90 to promote NF-κB translocation and cytokine production in GBM cells. 3. As a potential novel therapeutic agent for GBM, miR-101 modulated TIL accumulation by reversing LRRC4 promoter hypermethylation and induced LRRC4 re-expression in GBM cells.

## Results

### LRRC4 Inhibited the Infiltration of Ti-Treg Cells in GBM by Promoting Cytokines Secretion

**Figure 1:**
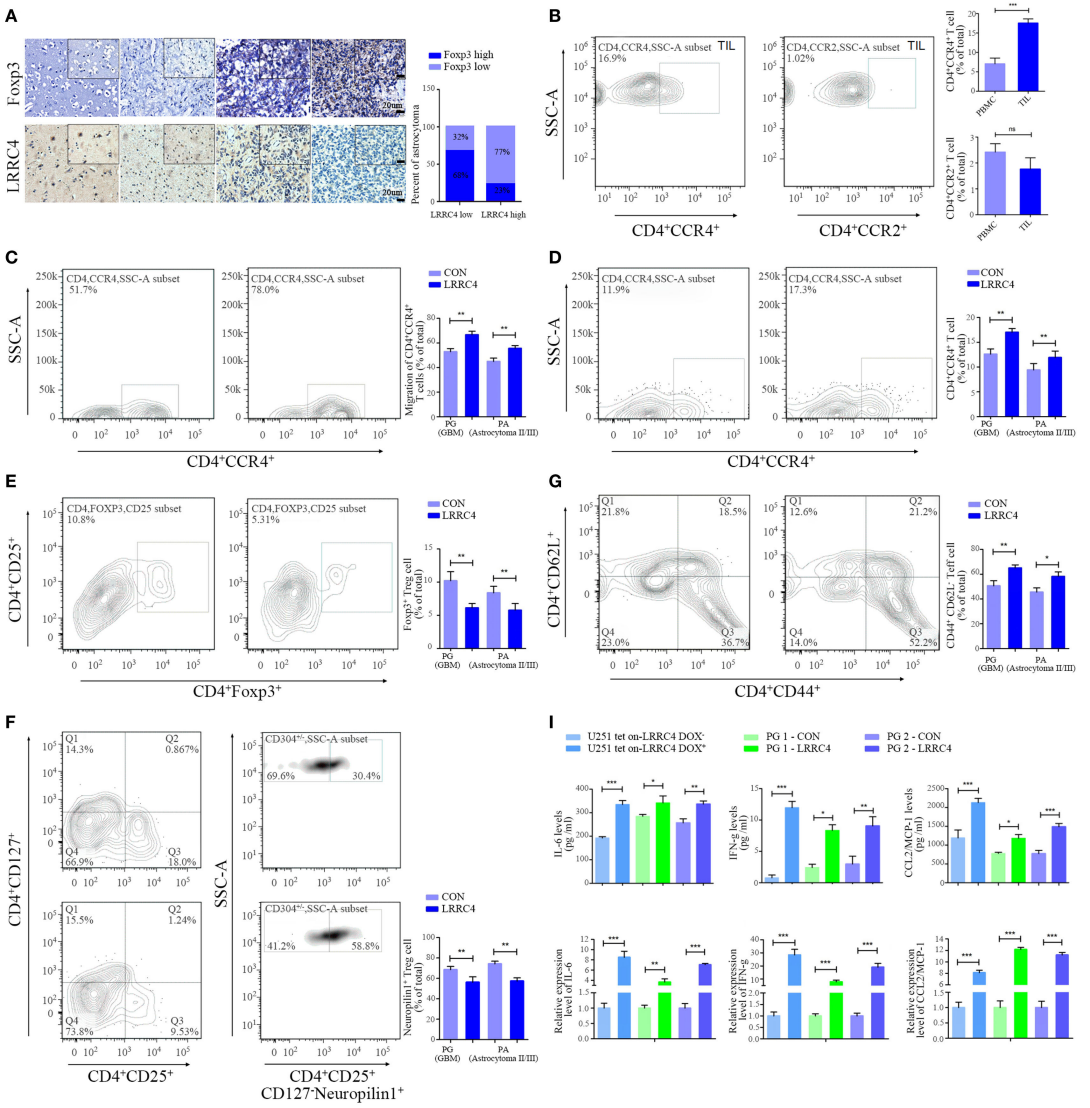

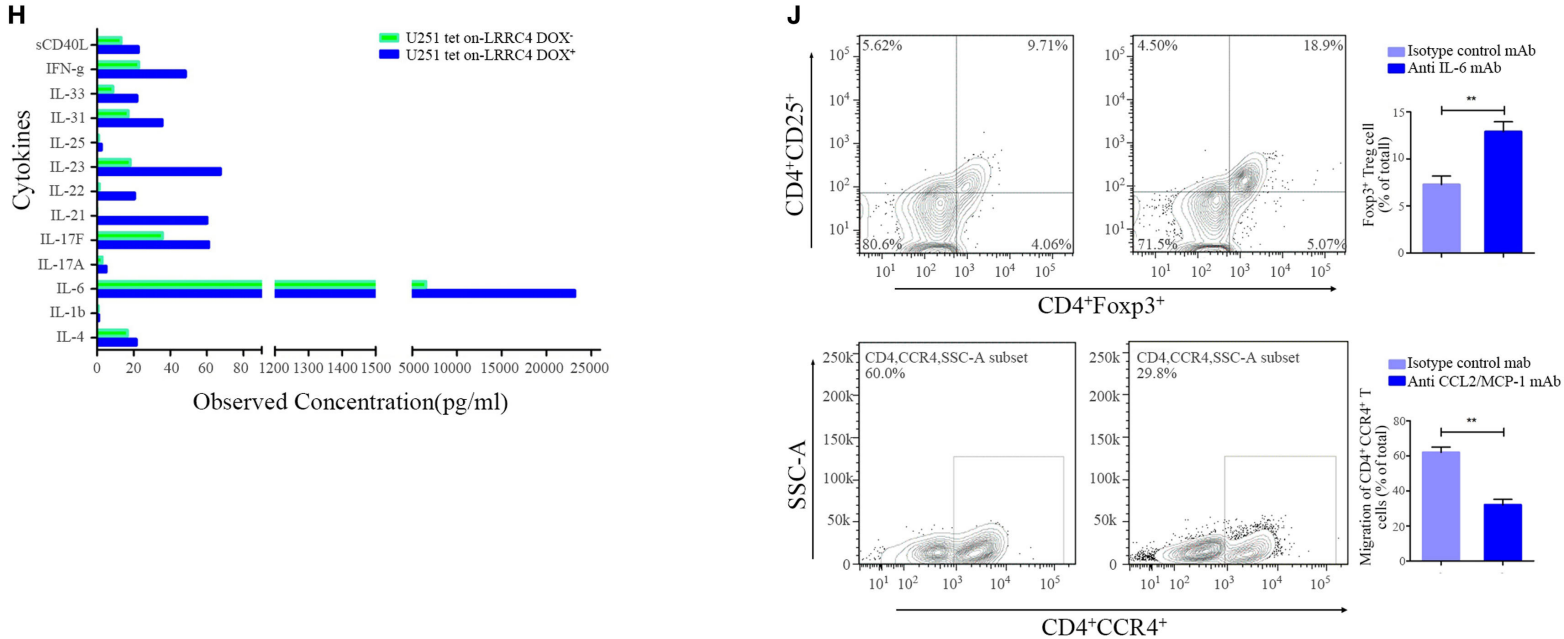
LRRC4 inhibited the infiltration of Ti-Treg cells in glioblastoma multiforme (GBM) by promoting the secretion of cytokines. **(A)** Immunohistochemistry analysis of Foxp3 and LRRC4 in normal brain (*n* = 7), grade II astrocytoma (*n* = 10), grade III astrocytoma (*n* = 10), and GBM tissues (*n* = 20). The average IHC score was recorded as low expression (score ≤ 8) or high expression (score > 8). **(B)** Fractions of CD4^+^CCR4^+^ T cells and CD4^+^CCR2^+^ T cells in tumor-infiltrating lymphocytes (TILs) and peripheral blood mononuclear cells (PBMCs) were evaluated by FACS analysis. CD4 population was gated (Figure S1 in Supplementary Material). CD4^+^CCR4^+^ T cells in TILs were higher than that in PBMC (***P < 0.001). There were no significant differences in the fractions of CD4^+^CCR2^+^ T cells (*P* > 0.05). The data summarize the results obtained for the TILs or PBMCs from nine GBM patients; the CD4 population was gated in all flow cytometry analyses in this study. **(C)** The conditioned medium from PG-LRRC4 and PA-LRRC4 cells induced much more CD4^+^CCR4^+^ T cell chemotaxis than the conditioned medium from PG-CON and PA-CON cells (**P < 0.01, primary GBM and astrocytoma cells transfected by pcDNA3.1-CON/LRRC4 were referred to simply as PG-CON/LRRC4 and PA-CON/LRRC4). **(D–G)**. The conditioned medium of PG-LRRC4 cells and PA-LRRC4 cells led to enhanced CD4^+^CCR4^+^ T cell expansion [**(D)**, **P < 0.01], reduced CD4^+^CD25^+^Fxop3^+^ regulatory T (Treg) cell expansion [**(E)**, **P < 0.01], mainly CD4^+^CD25^+^CD127^−^neuropilin1^−^ Ti-iTreg cells [**(F)**, **P < 0.01], and an increased percentage of CD4^+^CD44^+^CD62L^−^ Teff cells [**(G)**, **P < 0.01 and *P < 0.05] compared with the conditioned medium of PG-CON cells and PA-CON cells upon T-cell receptor stimulation (in the presence of anti-CD3/CD28 antibody and IL-2). The data summarize the results of TILs from 16 patient samples (9 WHO grade IV GBM patients, 5 WHO grade III grade, 2 WHO grade II grade). **(H)** The conditioned medium of U251Tet-on-LRRC4 DOX^+/−^ cells was harvested 24 h later and assayed for 13 different cytokine targets by Bio-Rad BioPlex analysis. **(I)** The expression and secretion of interleukin-6 (IL-6), interferon gamma (IFN-g), and CCL2 were increased in U251Tet-on-LRRC4 DOX^+^ and PG-LRRC4 cells (*P < 0.05, **P < 0.01, and ***P < 0.001). **(J)** The conditioned medium of U251-pcDNA3.1 LRRC4 cells mediating the chemotaxis of CD4^+^CCR4^+^ T cells could be blocked by anti-CCL2mAb; the conditioned medium of U251-pcDNA3.1 LRRC4 cells mediating the inhibition of Ti-Treg cells could be blocked by anti-IL-6 mAb (**P < 0.01).

As shown in Figure 1A, the expression absence of LRRC4 was accompanied by an increase in Foxp3^+^ Treg cell infiltration in GBM (Figure 1A). To test whether the recovery of LRRC4 expression in GBM cells could inhibit the infiltration of Treg cells in GBM tissues, primary cultured GBM cells, TILs, and peripheral blood mononuclear cells (PBMCs) from GBM patients were collected (Figure S1A and Table S1 in Supplementary Material). We found that tumor-infiltrating CD4^+^CCR4^+^ T cells, but not CD4^+^CCR2^+^ T cells, were enriched in GBM tissues (Figure S1A in Supplementary Material; Figure 1B), suggesting that CD4^+^CCR4^+^ T cells had relatively specific infiltration in GBM. The fraction of CD4^+^CCR4^+^ T cells in TILs was higher than that of PBMCs (Figure 1B). Furthermore, the conditioned medium from primary cultured GBM and astrocytoma cells transfected with pcDNA3.1 LRRC4 (hereafter referred to as PG-LRRC4 and PA-LRRC4, respectively, Figure S1B in Supplementary Material) induced much greater CD4^+^CCR4^+^ T cell chemotaxis and expansion than that from untransfected LRRC4 control cells (Figures 1C,D); however, the expansion of tumor-infiltrating CD4^+^CD25^+^Foxp3^+^ Treg cells (Ti-Treg) was inhibited (Figure 1E), especially the proportion of CD4^+^CD25^+^CD127^−^neuropilin^−^ Ti-iTreg cells (Figure 1F, CD127 expression inversely correlates with Foxp3 expression, and the combined use of CD4^+^, CD25^+^, and CD127^−^ can also define the Treg cell population with suppressive functions), but drove CD4^+^ tumor-infliltrating effector T cells (Ti-Teff) expansion (Figure 1G). Ti-Treg and Ti-Teff cells are the main subsets of CD4^+^CCR4^+^ T cells, and they always are recruited into the tumor microenvironment *via* the CCL2/CCR4 axis (12, 29–32). In this study, nine primary cultured astrocytoma cells were successfully gained in sixteen patient samples (seven cases were WHO grade IV GBM cells, one case was WHO grade III, one case was WHO grade II). Unfortunately, all of these cells were IDH1 wild type with a 1p/19q mutant status and loss of LRRC4 expression (Table S1 in Supplementary Material). The effect of LRRC4 of p53^wt^ and p53^mut^ PG cells on CD4^+^CCR4^+^ T cells showed a similar tendency. Subsequently, we detected the effect of the conditional medium derived from IDH1^wt^ U251 Tet-on-LRRC4 cells on CD4^+^CCR4^+^ T cells, Ti-Treg cells and Ti-Teff cells and obtained results that were consistent with those obtained for primary cultured GBM cells (Figures S1C–F in Supplementary Material). The above data indicated that LRRC4 promoted chemotaxis and accumulation of CD4^+^CCR4^+^ T cells, the LRRC4 deletion in GBM cells was one cause of the accumulation of Ti-Treg cells (mainly neuropilin^−^ Treg cells) in GBM, and re-expression of LRRC4 created a permissive intratumoral environment for Ti-Teff cell infiltration by inhibiting Ti-Treg cells. These effects were not correlated to the WHO grade or molecular typing of the astrocytoma (Figures 1C–G).

To investigate how Ti-Treg cell accumulation was inhibited by LRRC4, we measured the levels of 13 cytokines in the conditioned medium of U251 Tet-on-LRRC4 cells by Bioplex array analysis and found that re-expression of LRRC4 promoted the secretion of 9 cytokines (Figure 1H). In doxycycline (DOX)-induced U251 Tet-on-LRRC4 cells and PG-LRRC4 GBM cells, LRRC4 resulted in increased expression and secretion of interleukin-6 (IL-6) and interferon gamma (IFN-g) (Figure 1I). As mentioned previously, CCR4 receptors bind to the chemokine CCL2 produced by GBM cells and mediate the chemotaxis of T cells (32). LRRC4 also promotes the expression and secretion of CCL2 in GBM cells (Figure 1I). To verify whether these cytokines were responsible for the modulation of LRRC4 on T cells, anti-IL-6/CCL2 antibody were used, and we found that anti-IL-6 antibody and anti-CCL2 antibody could block the inhibition of Ti-Treg cell expansion and the promotion of CD4^+^CCR4^+^ T cell chemotaxis by LRRC4, respectively (Figure 1J). These results indicated that LRRC4 suppressed the accumulation of Ti-Treg cells and increased the expansion of Ti-Teff cells by promoting GBM cell cytokine secretion.

### LRRC4 Inhibited the Infiltration of Ti-Treg Cells *via* GBM Cell-Derived Cytokine-Free and PD-1-Containing Exosomes

**Figure 2:**
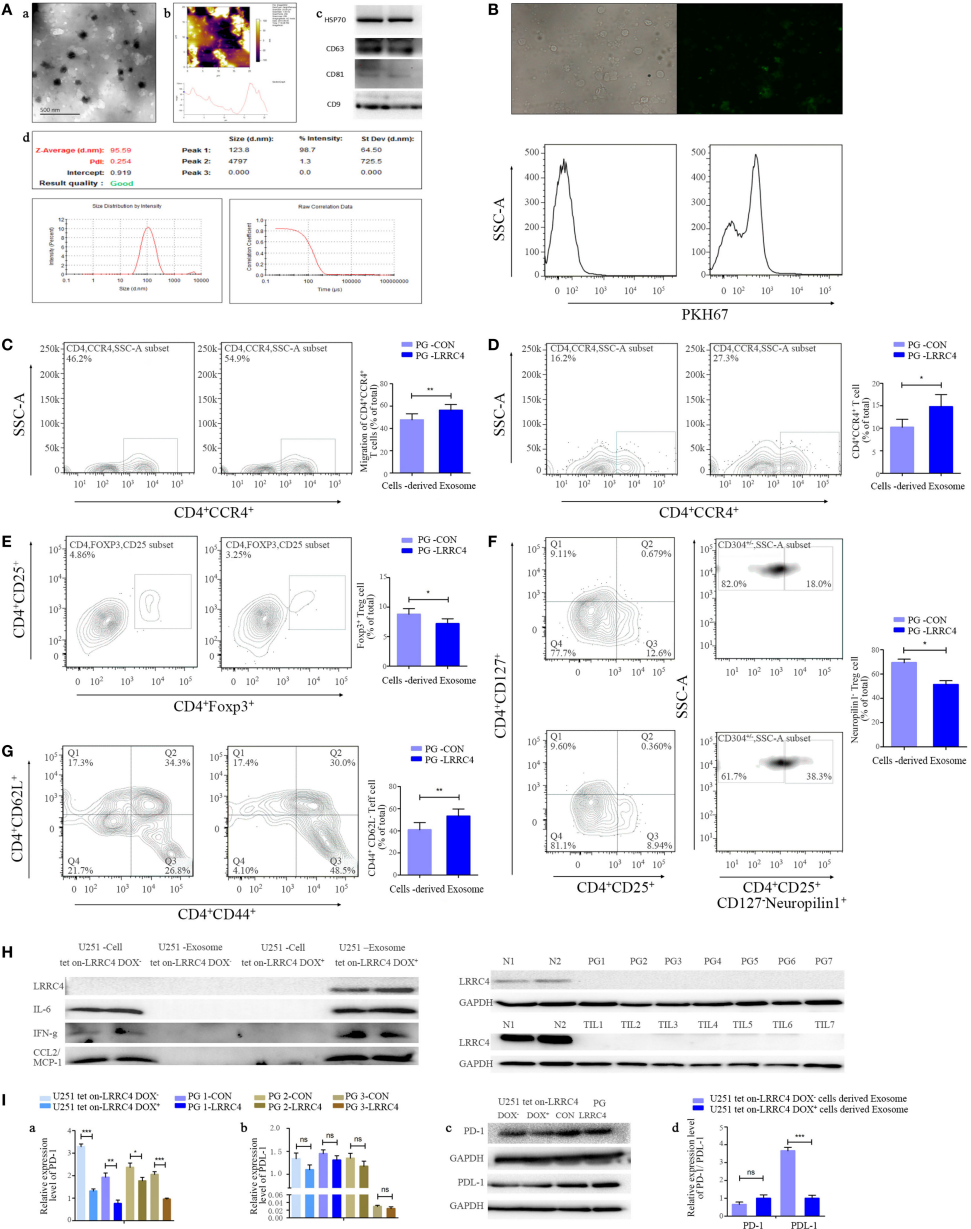
LRRC4 inhibited the infiltration of Ti-Treg cells by glioblastoma multiforme (GBM) cell-derived cytokine-free and programmed cell death 1 (PD-1)-containing exosomes. [**(A)**, a,b] Transmission electron and atomic force microscopy micrographs of exosomes (isolated from the conditioned medium of GBM cells) revealing the typical morphology and size. (c) The published exosomal markers CD63, CD81, HSP70, and CD9 were detected. (d) The particle size distribution of EVs was measured using the ZetaView^®^ Particle Tracking Analyzer. **(B)** GBM cell-derived exosomes were stained with PKH67 (green) and incubated with tumor-infiltrating lymphocytes (TILs). TILs were visualized using an immunofluorescence microscope (upper panel) and FACS analysis (lower panel). **(C)** PG-LRRC4 and PA-LRRC4 cell-derived exosomes induced much more CD4^+^CCR4^+^ T cell chemotaxis than PG-CON and PA-CON cell-derived exosomes (**P < 0.01). **(D–G)** PG-LRRC4 cell-derived exosomes led to enhanced CD4^+^CCR4^+^ T cell expansion [**(D)**, *P < 0.05], reduced CD4^+^CD25^+^Fxop3^+^ regulatory T (Treg) cell expansion, especially the expansion of CD4^+^CD25^+^CD127^−^neuropilin1^−^ Ti-iTreg cells [**(E,F)** *P < 0.05], and an increased percentage of CD4^+^CD44^+^CD62L^−^ Teff cells [**(G)**, **P < 0.01] compared with PG-CON cell-derived exosomes; TILs isolated from GBM tissues were seeded in 48-well plates, and co-incubated with 20 μg/ml GBM cells-derived exosomes under anti-CD3/CD28 conditions. After coculturing 3 days (72 h), TILs were harvested, and CD4^+^CCR4^+^ T cells, Treg cells, and Teff cells were analyzed by FACS. **(C–G)**. Data summarizing the results obtained for TILs generated from seven GBM patients. **(H)** LRRC4, interleukin-6 (IL-6), CCL2, and interferon gamma (IFN-g) protein levels were absent in U251 tet-on-LRRC4 DOX^+/−^ cell-derived exosomes (upper panel), and no LRRC4 expression was found in PG cells from seven cases; matched TILs and normal brain tissues were from two cases (N1 and N2, lower panel). **(I)** PD-1 (a,c), but not PDL-1 (b,c), levels were increased in U251 Tet-on-LRRC4 DOX^+^ and PG-LRRC4 cells compared with U251 Tet-on-LRRC4 DOX^−^ cells and PG-CON cells (*P < 0.05, **P < 0.01, and ***P < 0.001). (d) The expression of PDL-1, but not PD-1, was increased in U251 Tet-on-LRRC4 DOX^+^ cell-derived exosomes compared with U251 Tet-on-LRRC4 DOX^−^ cell-derived exosomes (*P < 0.001).

Exosomes serve as a signaling carrier mediating tumor cell and T cell communication (33–39). To test whether LRRC4 affected the communication between GBM cells and CD4^+^CCR4^+^ T cells through exosomes, we isolated exosomes from the conditioned medium of U251 Tet-on-LRRC4 and PG-LRRC4/CON cells (Figure 2A) and verified that these exosomes were transmitted into TILs (Figure 2B). The exosomes derived from LRRC4 overexpression GBM cells caused significant chemotaxis and expansion of CD4^+^CCR4^+^ T cells (Figures 2C,D), inhibited the proportion of Ti-Treg cells (Figure 2E), mainly the CD4^+^CD25^+^CD127^−^neuropilin^−^ Ti-iTreg cells (Figure 2F), and promoted Ti-Teff cell expansion (Figure 2G), consistent with the results obtained using the conditioned medium. However, these exosomes only slightly reduced the expansion of Ti-Treg cells, and we did not detect LRRC4 expression in the exosomes or TILs (Figure 2H). Simultaneously, IL-6, IFN-g, and CCL2 were not transported by exosomes (Figure 2H), suggesting that LRRC4 was not transferred into TILs from GBM cells through exosomes but mainly exerted its inhibitory function on Ti-Treg cell expansion by directly promoting cytokine secretion into the conditioned medium of GBM cells.

The kinds of molecules that will be transferred into recipient cells through exosomes to exert vital biological functions in the presence of LRRC4 in GBM cells are unknown. PD-1 is an important target that is applied to melanoma clinical therapy, and the expression of PD-1 and its ligand PDL-1 in Treg cells or tumor cells protects tumors from immune-mediated rejection and promotes tumor progression (15, 16, 40). We found that PD-1 and PDL-1 were expressed in GBM cells (Figure 2Ia–c) and LRRC4 decreased the level of PD-1 in GBM cells (Figure 2Ia–c); however, it did not affect expression in the exosomes (Figure 2Id). Interestingly, although LRRC4 did not alter PDL-1 expression in GBM cells (Figure 2Ib,c), LRRC4 inhibited PDL-1 that was packed into exosomes (Figure 2Id) and reduced the transmission of PDL-1 from GBM cells to TILs. Thus, LRRC4 also regulated Ti-Treg cells and Ti-Teff cells through cytokine-free and PDL-1-containing exosomes.

### LRRC4 Activated NF-κB Signaling by Binding to PDPK1, and HSP90 Is Required for the Interaction of LRRC4 and PDPK1

**Figure 3:**
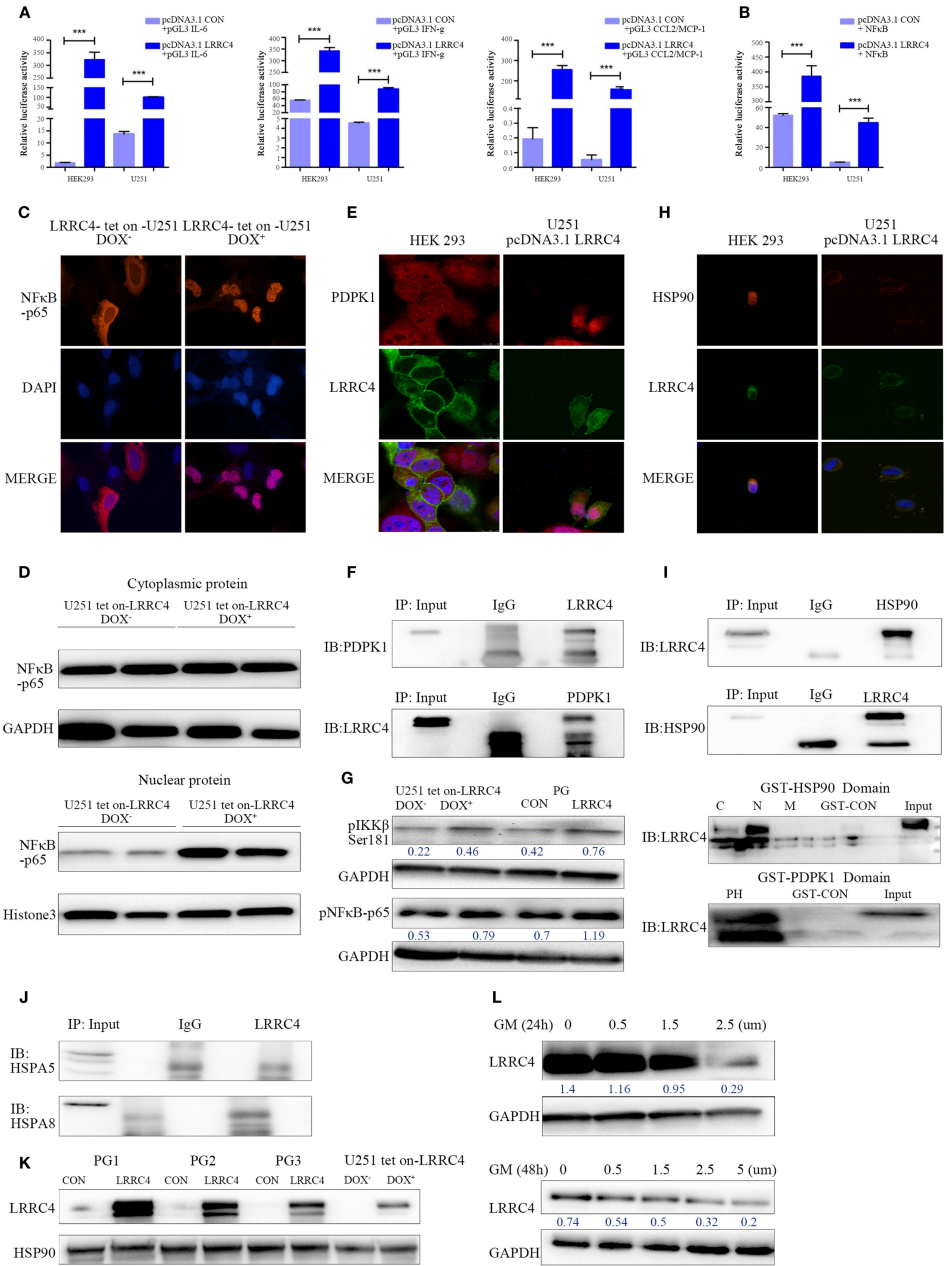
LRRC4 facilitated IKKβ/NF-κB pathway activation by binding to phosphoinositide dependent protein kinase 1 (PDPK1), and HSP90 was required for the interaction of LRRC4 and PDPK1. **(A,B)** The Dual-Luciferase Reporter Assay indicated that LRRC4 promoted the transcriptional activity of interleukin-6 (IL-6) (***P < 0.001), interferon gamma (IFN-g) (***P < 0.001) and CCL2 [**(A)**, P*** < 0.001], and induced NF-κB activation [**(B)**, ***P < 0.001]. **(C)** Immunoflorescent staining for NF-κB-p65 revealed the nucleus translocation in U251 Tet-on-LRRC4 DOX^+^ cells compared with U251 Tet-on-LRRC4 DOX^−^ cells. **(D)** The expression of NF-κB-p65 was mainly detected in the nucleus in U251 Tet-on-LRRC4 DOX^+^ cells. **(E)** Representative confocal and immunofluorescence images showing the co-localization of LRRC4 (green) and PDPK1 (red) in the cytoplasm in HEK293 and U251-pcDNA 3.1 LRRC4 cells. **(F)** Co-IP analysis showing the interaction between LRRC4 and PDPK1 in HEK293 and U251-pcDNA 3.1 LRRC4 cells. **(G)** Western blot analysis showing that the expression of pIKKβ^Ser181^ and pNF-κB p65 was increased in U251 Tet-on-LRRC4 DOX^+^ cells and PG-LRRC4 cells. **(H)** Representative confocal and immunofluorescence images showing the co-localization of LRRC4 (green) and HSP90 (red) in the cytoplasm in HEK293 and U251-pcDNA 3.1 LRRC4 cells. **(I)** Co-IP analysis showing the interaction between LRRC4 and HSP90 in U251-pcDNA 3.1 LRRC4 cells (up); GST pull-down assay showing that LRRC4 mainly bound to the N-domain of HSP90; LRRC4 mainly bound to the PH domain of PDPK1 (down). **(J)** Co-IP analysis showing no interaction between LRRC4 and HSPA5 and HSPA8 in U251-pcDNA 3.1 LRRC4 cells. **(K)** Western blot analysis of the levels of HSP90 showing no alteration in U251 Tet-on-LRRC4 DOX cells and PG-CON/LRRC4 cells. **(L)** The expression of LRRC4 was decreased when HSP90 activity was inhibited. The U251-pcDNA 3.1 LRRC4 cells were treated with selected concentrations and durations of the HSP90 inhibitor geldanamycin.

LRRC4 inhibited Ti-Treg cell accumulation and promoted Ti-Teff cell expansion through cytokines in GBM, but the underlying mechanism remains unclear. We observed that LRRC4 increased the luciferase activity of IL-6, IFN-g, and CCL2 in HEK293 and GBM cells, respectively (Figure 3A). A number of reports have shown that NF-κB regulates the production of various kinds of cytokine in immune cells as well as tumor cells. LRRC4 elevated the luciferase activity of NF-κB (Figure 3B) and promoted its translocation into the nucleus in DOX-induced U251 Tet-on-LRRC4 cells (Figures 3C,D). Thus, we questioned how LRRC4 induced the nuclear translocation of NF-κB. 3-PDPK1, a key regulator of NF-κB activation (15, 16, 40), was a potential binding protein of LRRC4 (predicted by scancite 2.0 software). PDPK1 promotes IKKβ/NF-κB signaling activation and nuclear translocation by directly phosphorylating IKKβ at the Ser181 residue (41). We first confirmed that LRRC4 and PDPK1 were co-localized and interacted in the cytoplasm in HEK293 and U251 cells (Figures 3E,F). We further found that LRRC4 could upregulate the phosphorylation of IKKβ^Ser181^ and pNF-κBp65 (Figure 3G). Previous literature has reported that the stability and signaling of PDPK1 is protected by the molecular chaperone HSP90 (heat shock protein 90) (42), and inhibition of HSP90 results in PDPK1 depletion and thus triggers a cascade of NF-κB deactivation. Our study first verified that LRRC4 and HSP90 were co-localized and interacted in the cytoplasm (Figures 3H,I), and LRRC4 mainly bound to the N-domain of HSP90 and the PH domain of PDPK1 (Figure 3I). In addition to HSP90, HSP70 family members also act as molecular chaperones, but LRRC4 did not interact with HSPA8 and HSPA5 (Figure 3J). LRRC4 did not affect the expression of HSP90 (Figure 3K), but the HSP90 inhibitor geldanamycin blocked the re-expression of LRRC4 in GBM cells (Figure 3L). These results suggested that HSP90 was also a molecular chaperone of LRRC4 only in the presence of HSP90, and LRRC4 interacted with PDPK1 to activate NF-κB and promote its nuclear translocation.

### miR-101 Reversed LRRC4 Hypermethylation to Induce LRRC4 Re-Expression in GBM Cells

**Figure 4:**
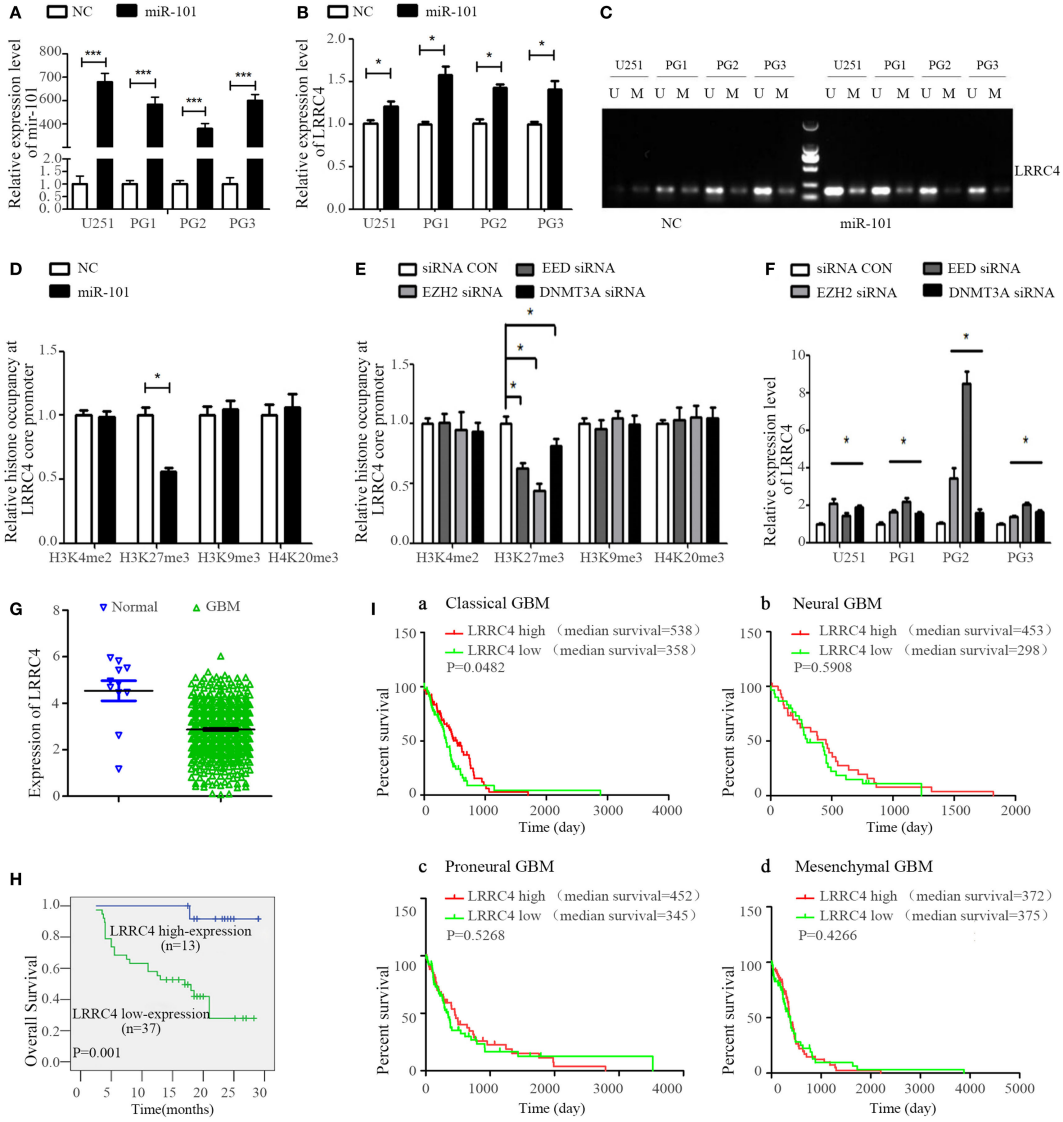
miR-101 reversed the hypermethylation of LRRC4 and induced LRRC4 re-expression in glioblastoma multiforme (GBM) cells by targeting EZH2, EED, and DNMT3A. **(A)** The miR-101 level was increased after GBM cells were transfected with miR-101/NC (*P < 0.001). **(B)** miR-101 promoted LRRC4 mRNA expression (*P < 0.05). **(C)** miR-101 reversed the hypermethylation status of LRRC4 by MSP in GBM cells; U, unmethylated primer; M, methylated primer. **(D)** H3K27me3 occupancy of the LRRC4 core promoter was inhibited by miR-101. A ChIP assay was used to detect the H3K4me2, H3K27me3, H3K9me3, and H4K20me3 occupancy of the LRRC4 core promoter (*P < 0.05). **(E)** H3K27me3 occupancy of the LRRC4 core promoter was inhibited by EZH2 siRNA, EED siRNA, and DNMT3A siRNA. A ChIP assay was performed to detect the H3K4me2, H3K27me3, H3K9me3, and H4K20me3 occupancy of the LRRC4 core promoter. GBM cells were transfected with EZH2 siRNA, EED siRNA, and DNMT3A siRNA (*P < 0.05). **(F)** LRRC4 expression was increased after GBM cells were transfected with EZH2 siRNA, EED siRNA, DNMT3A siRNA, or a siRNA NC (*P < 0.05). **(G)** LRRC4 expression in GBMs and normal brain tissues from the Cancer Genome Atlas (TCGA). **(H)** Kaplan–Meier curve analysis indicated that the high LRRC4 expression was correlated with a better survival prognosis in astrocytoma patients (*n* = 50). **(I)** Kaplan–Meier curve analysis in four subtypes of TCGA GBM patients (classical, neural, proneural, and mesenchymal) stratified by LRRC4 expression. Classical subtype GBM patients with high LRRC4 expression had a better overall survival, and the median OS was higher in high LRRC4 expression than in low LRRC4 expression patients with neural and proneural GBM.

LRRC4 has been verified to be a hypermethylated gene and loss expression in glioblastoma (20, 28). miR-101 is a suppressor gene for glioma. Re-expression of miRNA-101 could increase LRRC4 expression (Figures 4A,B) and reverse the LRRC4 hypermethylation status in GBM cells (Figure 4C), but LRRC4 is not the direct target gene of miR-101 (Figure S1H in Supplementary Material). Histone methylation modification and DNA methyltransferase are critical factors that regulate gene promoter methylation. Our previous study found that miR-101 targets DNMT3A, EED, and EZH2 directly and decreases DNMT3A, EED, and EZH2 expression (43–46). Hypermethylation was one of the mechanisms of LRRC4 loss in GBM, and therefore we speculated that miR-101 promoted LRRC4 expression in a methylation- or histone- correlated pattern. Previous studies have confirmed the methylation of K4, K9, and K27 on histone H3. H3K4 methylation typically activates the gene promoter, whereas H3K20 and H4K27 methylation repress the gene promoter. H3K9 trimethylation can repress or activate the gene promoter, and H3K9 dimethylation represses the gene promoter (47). Here, we found that miR-101 decreased H3K27me3 enrichment on the LRRC4 core promoter [details of the LRRC4 core promoter can be found in our previous study (28)] but have no effect on H3K4me2, H3K9me3, and H4K20me3 enrichment of the LRRC4 core promoter (Figure 4D), which was consistent with the effects of EZH2, EED, and DNMT3A inhibitors on histone modifications in the core promoter region of LRRC4 (Figure 4E). We also found that EZH2, EED, and DNMT3A inhibitors upregulated LRRC4 expression in GBM cells (Figure 4F). These data indicated that miR-101 reversed the hypermethylation of LRRC4 promoter and induced the re-expression of LRRC4 in GBM cells by directly targeting EZH2, EED, and DNMT3A to decrease H3K27me3 enrichment of the LRRC4 core promoter.

In addition, we found that LRRC4 expression was closely correlated with the prognosis of astrocytoma patients, with high expression of LRRC4 suggesting a good prognosis (Figures 4G,H). Moreover, the Cancer Genome Atlas data analysis further indicated that classical subtype GBM patients with high LRRC4 expression had the best prognosis, and neural and proneural subtype GBM patients with high LRRC4 expression had a longer median survival time than the patients with low LRRC4 expression (Figure 4I).

### miR-101 Inhibited Ti-Treg Cell Infiltration in GBM by Epigenetically Targeting LRRC4

**Figure 5:**
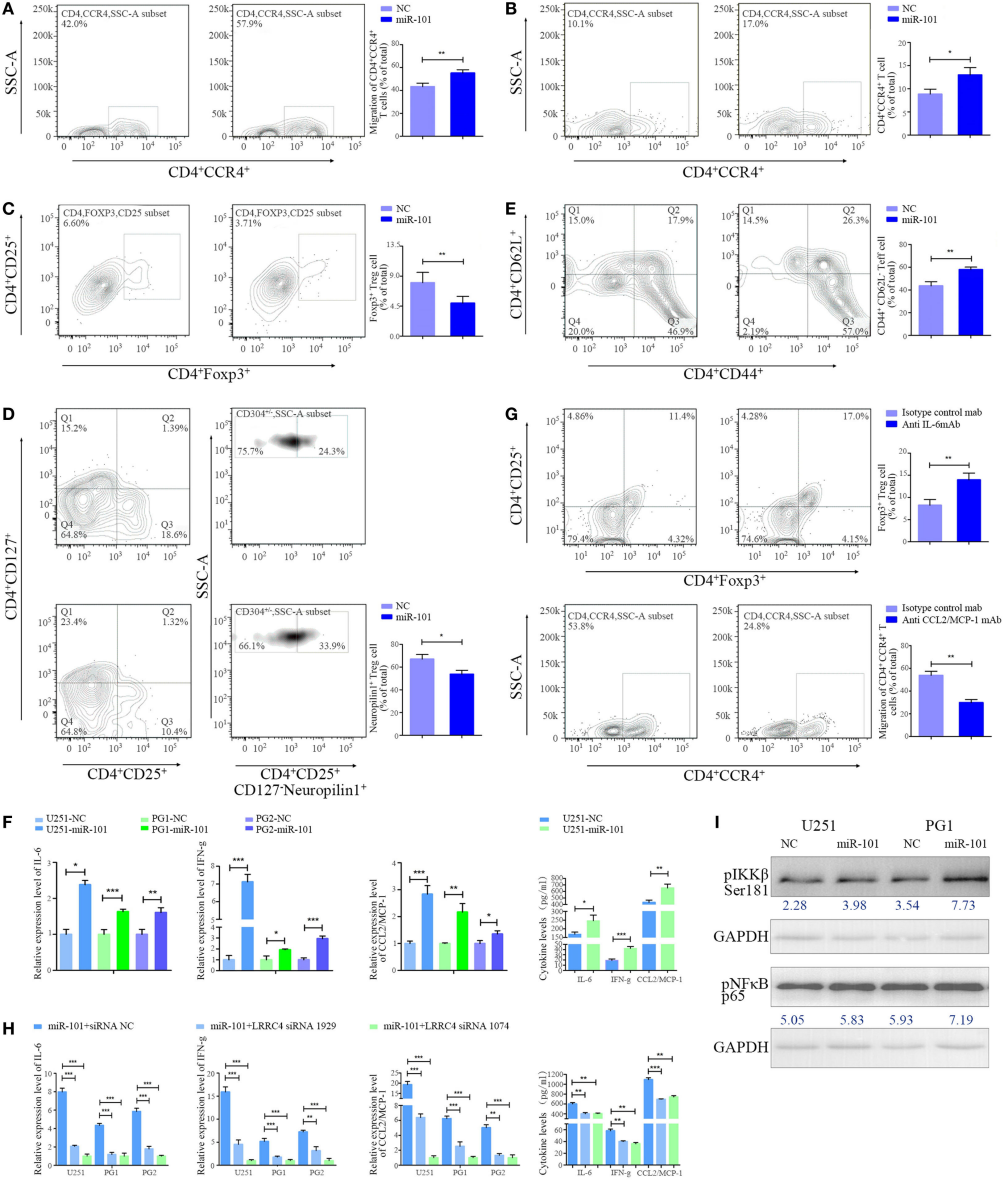
miR-101 inhibited Ti-Treg cell expansion by restoring LRRC4 expression. **(A)** The conditioned medium from miR-101 overexpression glioblastoma multiforme (GBM) cells induced much more CD4^+^CCR4^+^ T cell chemotaxis than the conditioned medium from control cells (**P < 0.01). **(B–E)** The conditioned medium from miR-101 overexpression GBM cells led to enhanced CD4^+^CCR4^+^ T cell expansion [**(B)**, *P < 0.05], reduced CD4^+^CD25^+^Fxop3^+^ regulatory T (Treg) cell expansion [**(C)**, **P < 0.01], mainly neuropilin1^−^ Ti-iTreg cells [**(D)**, *P < 0.05], and an increased percentage of CD44^+^CD62L^−^ Teff cells [**(E)**, **P < 0.01] compared with the control cell conditioned medium upon T-cell receptor stimulation (in the presence of anti-CD3/CD28 antibody and IL-2); the data in panels **(B–E)** summarize the results obtained for TILs from seven GBM patients. **(F)** miR-101 facilitated the expression and secretion of interleukin-6 (IL-6), interferon gamma (IFN-g), and CCL2 in GBM cells (*P < 0.05, **P < 0.01, and ***P < 0.001). [**(G)**, upper] The conditioned medium of miR-101 overexpression GBM cells mediating the inhibition of Ti-Treg cells could be blocked by anti-IL-6 mAb (**P < 0.01). (lower) The conditioned medium of miR-101 overexpression GBM cells mediating the chemotaxis of CD4^+^CCR4^+^ T cells could be blocked by anti-CCL2 mAb (**P < 0.01). **(H)** LRRC4 siRNA 1074 and siRNA 1929 blocked the high level of expression and secretion of IL-6, IFN-g, and CCL2 induced by miR-101 (*P < 0.05, **P < 0.01, and ***P < 0.001). **(I)** The expression of pIKKβ^Ser181^ and pNF-κB p65 was increased in GBM cells transfected with miR-101.

We wondered whether miR-101 is capable of reversing suppressor gene LRRC4 re-expression in GBM cells and thus whether it could act as a potential drug to improve the immune microenvironment of GBM. The conditioned medium from miR-101-transfected GBM cells was obtained and cocultured with TILs. We found that the conditioned medium of miR-101 overexpressed (hereafter referred to as miR-101^OV^) GBM cells induced more chemotaxis and expansion of CD4^+^CCR4^+^ T cells than miR-101^CON^ cells (Figures 5A,B). miR-101 inhibited the expansion of Ti-Treg cells (Figure 5C), especially the proportion of CD4^+^CD25^+^CD127^−^neuropilin^−^ Ti-iTreg cells (Figure 5D), and drove Ti-Teff cell expansion (Figure 5E). In addition, we showed that miR-101 significantly increased the expression and secretion of IL-6, IFN-g, and CCL2 (Figure 5F), and the modulation of miR-101 on CD4^+^CD25^+^Foxp3^+^ Ti-Treg cells and CD4^+^CCR4^+^ T cells could be blocked by anti-IL-6 mAb (Figure 5Ga) and anti-CCL2 mAb (Figure 5Gb), respectively. Knockdown of LRRC4 blocked the high expression of cytokines induced by miR-101 (Figure 5H). In addition, miR-101 only slightly upregulated the phosphorylation of IKKβ^Ser181^ and pNF-κBp65, suggesting that miR-101 regulation of the IKKβ/NF-κB pathway likely consisted of more complex mechanisms (Figure 5I). These results demonstrated that miR-101 mediated cytokine secretion to modulate T cell accumulation by epigenetically targeting LRRC4 of GBM cells. miR-101 may be as a potential drug to modulate GBM cells and TILs in GBM.

## Discussion

Glioblastoma multiforme is a complex tumor consisting of tumor and non-tumor cells, each of which is individually conducive to GBM development and treatment response. Most of the non-tumor cells are tumor-infiltrating T cells and microglia, among others, that create a supportive stroma microenvironment for tumor cell growth and invasion (2–4). Mounting evidence is emerging that tumor-infiltrating T cells exert a critical role in the GBM microenvironment, but there is little information concerning the interaction of GBM cells and tumor-infiltrating T cells in brain tumors. Previous studies have shown that CCR4^+^ T cells can be recruited to the GBM microenvironment (Ti-CCR4^+^ T cells) (12, 32), and our data further indicated that CD4^+^CCR4^+^ T cells were relatively specifically enriched in GBM and more abundant in the tumor microenvironment than those in the peripheral blood.

LRRC4 is a tumor suppressor gene for glioma (20). Our research showed that re-expression of the suppressor gene LRRC4 in GBM cells mediated the interaction between GBM cells and tumor-infiltrating T cells. Re-expression of LRRC4 in GBM cells enhanced chemotaxis of CD4^+^CCR4^+^ T cells by promoting CCL2 production, and reports have confirmed that CCL2 contributes to CD4^+^CCR4^+^ T cell accumulation in GBM. We also showed that Treg cell infiltration in the tumor microenvironment increased with the astrocytomas grade and that almost all Ti-Treg cells expressed the CCR4 receptor, whereas LRRC4 expression showed a negative correction with Ti-Treg cells in GBM. Interestingly, re-expression of LRRC4 in GBM cells stimulated the accumulation of CD4^+^CCR4^+^ T cells but simultaneously inhibited CCR4^+^ Ti-Treg cell expansion and facilitated Ti-Teff cell expansion. This finding implied that LRRC4 may play a dual role to construct a positive immune microenvironment through Ti-Treg inhibition and Teff cell promotion. Simultaneously, this phenomenon probably explained why the expression of LRRC4 in GBM cells inhibited the expansion of CCR4^+^ Treg cells, but the proportion of CD4^+^CCR4^+^ T cells continued to increase. Our data further demonstrated that LRRC4 significantly facilitated IL-6 production by GBM cells and mediated the interaction between GBM cells and Ti-Treg cells *via* IL-6. Recent studies have shown that IL-6 induces the differentiation of naïve T cells into Teff cells but not Treg cells, polarizes Treg cells to adopt a Teff cell phenotype and blocks the suppressor activity of Treg cells (13, 48, 49). These functions of IL-6 probably explain the ability of LRRC4 in GBM cells to inhibit Ti-Treg cells. Collectively, our results showed that re-expression of LRRC4 in GBM cells modulated the GBM microenvironment through multiple cytokines.

In addition to cytokines, exosomes also serve as signaling tools mediating tumor cell and T cell communication. For example, in nasopharyngeal carcinoma, colorectal cancer, and lung cancer, tumor cell-derived exosomes modulate Treg cell expansion (50–52). Exosomes secreted by cancer cells involving glioma cells have been shown to express immunosuppressive molecules such as PDL-1 or PD-1 (15, 16, 40). PD-1 is a checkpoint receptor that, upon engagement by PDL-1, dampens Teff cell functions by inhibiting T-cell receptor signaling. The PDL-1–PD1 axis induces apoptosis or exhaustion of activated immune cells, converting naive T cells, and TH cells into Treg cells, and the constitutive expression of PDL-1 and PD-1 on Treg cells regulates the formation of stable and productive immunological contacts, providing a novel mechanism of suppression that is utilized by Treg cells. Furthermore, PD-1 is also expressed in several types of cancer (including glioma, breast cancer, lung cancer, among others) and promotes cancer progression. Thus, the PDL-1–PD1 axis protects tumor from immune cell-mediated rejection, and exosomes (containing PD-1 and/or PDL-1) immune suppression is associated in some situations with a tumor-suppressive microenvironment (15, 16, 40). In this study, we also verified the critical role of exosomes in the modulation of the GBM microenvironment. Exosomes derived from LRRC4-recovered GBM cells caused a significant chemotaxis and expansion of CD4^+^CCR4^+^ T cells facilitated Ti-Teff cell expansion and inhibited the proportion of Ti-Treg cells. In contrast to previous reports, we found that PDL-1 and PD-1 were expressed together in GBM cells, and GBM cell-derived exosomes contained both PDL-1 and PD-1, with much lower levels of PD-1 than PDL-1. Interestingly, the recovery of LRRC4 in GBM cells only inhibited PDL-1 packing into exosomes and reduced the transmission of the ligand PDL-1 in GBM cells. PDL-1/PD-1 pathway from ligand and receptor aspects attenuated by LRRC4 contributed to the modulation of LRRC4-recovery GBM cells-derived exosomes on immune cells. We also found that LRRC4 in GBM cells was not transferred into TILs through exosomes, and cytokines (IL-1, IFN-g, and CCL2) were simply directly secreted into the conditioned medium.

In the future, multi omics analysis will be implemented in exosomes, and we will further focus on the contribution of the PD-1/PDL-1 pathway using molecular imaging and PD-1/PDL-1 knockdown/overexpression in exosomes to reveal the mechanisms of LRRC4 in GBM microenvironment immune cells modulation through exosomes.

As described earlier, re-expression of LRRC4 in GBM cells modulated CD4^+^CCR4^+^ T cells through multiple cytokines, but the underlying mechanism remains unclear. It is well known that NF-κB plays a critical role in inflammation and is a transcription factor for a number of immune-related genes (IL-6, CCL2, among others) involved in immune responses; thus, it plays a dual role in promoting and inhibiting cancer (53). It is also clear that PDPK1 is a kinase that phosphorylates several members of the protein kinase A, G, and C family, and PDPK1 directly phosphorylate IKKβ to activate NF-κB signaling (41). Our studies have revealed that LRRC4 is a novel regulator of PDPK1/NF-κB signaling. LRRC4 directly binds to PDPK1 in the presence of the molecular chaperone HSP90 and facilitates IKKβ^ser181^ and NF-κBp65 phosphorylation, promotes NF-κB nuclear translocation and activates NF-κB signaling to facilitate IL-6, CCL2, and IFN-g transcript and secretion. Overall, we show that LRRC4 exerts its inhibitory function on CD4^+^CCR4^+^ T cells in the GBM microenvironment *via* the PDPK1/IKKβ/NF-κB/cytokine pathway, and our findings provide new insights into PDPK1/NF-κB signaling.

**Figure 6:**
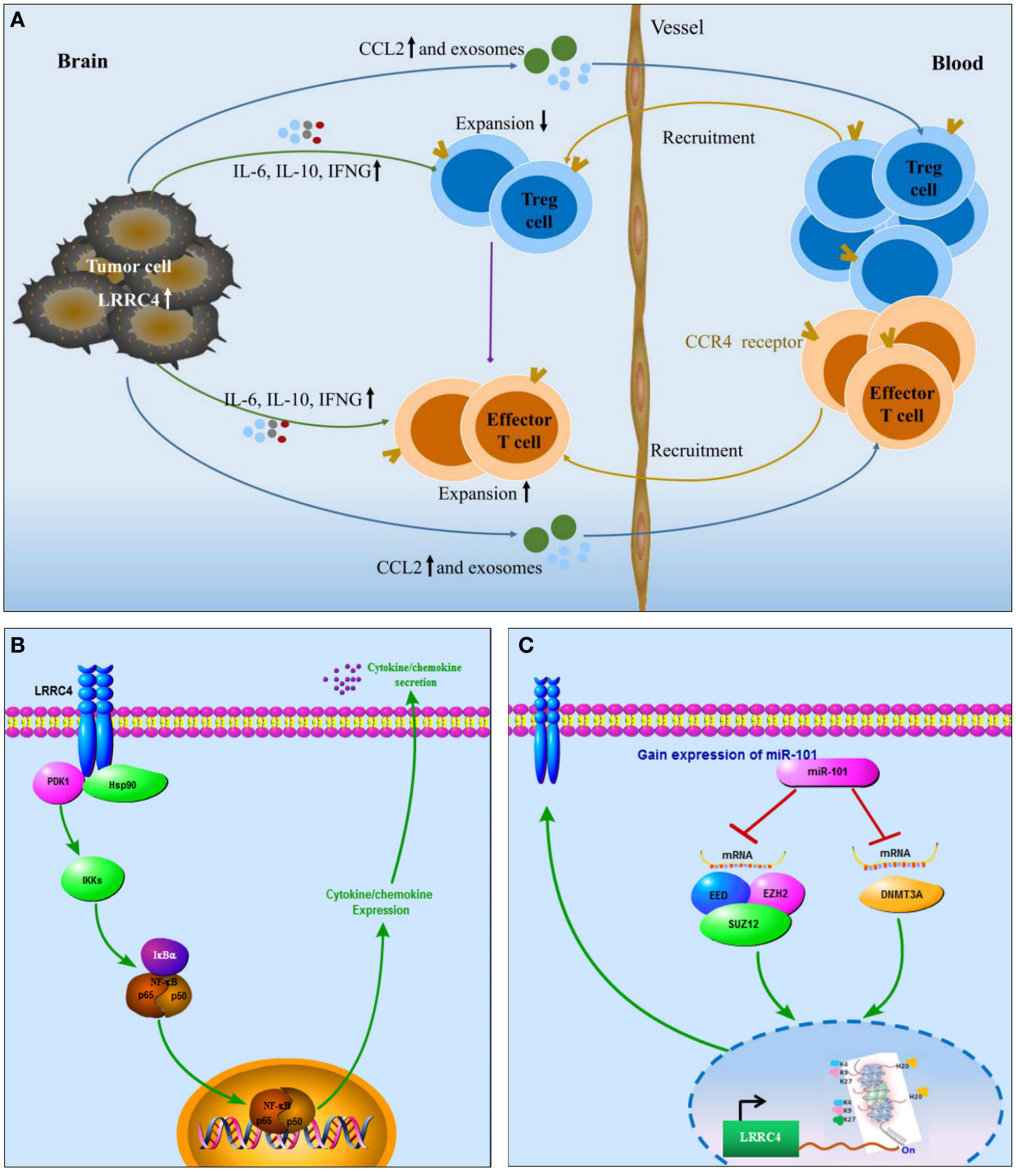
Schematic diagram. **(A)** LRRC4 mediated the interaction between glioblastoma multiforme (GBM) cells and CD4^+^CCR4^+^ T cells by cytokines and exosomes: LRRC4 re-expression in GBM cells recruits CD4^+^CCR4^+^ T cells involving regulatory T (Treg) cells and Teff cells into the GBM microenvironment through CCL2 and exosomes, while LRRC4 re-expression in GBM cells can inhibit CD4^+^CD25^+^Foxp3^+^ tumor-infiltrating Treg cells (CD4^+^CD25^+^Foxp3 Ti-Treg) expansion and facilitate tumor-infiltrating Teff (Ti-Teff) cell expansion by interleukin-6 (IL-6), interferon gamma (IFN-g), and exosomes (cytokine-free and programmed cell death 1-containing) secretion. The inhibition of Ti-Treg cells is also responsible for the expansion of Ti-Teff cells. **(B)** LRRC4 interacted with phosphoinositide dependent protein kinase 1 (PDPK1) and HSP90 to promote NF-κB nuclear translocation and cytokine secretion in GBM cells: as a novel regulator of PDPK1/NF-κB signaling, LRRC4 bound to PDPK1 in the presence of the molecular chaperone HSP90, increased IKKβ^ser181^ phosphorylation and NF-κB nuclear translocation, and activated NF-κB signaling to facilitate the expression and secretion of IL-6, CCL2, and IFN-g. **(C)** miR-101 reversed LRRC4 hypermethylation to induce LRRC4 re-expression: miR-101 reversed the hypermethylation of LRRC4 by directly targeting EZH2, EED, and DNMT3A to decrease H3K27me3 enrichment of the LRRC4 core promoter.

miR-101 is a tumor suppressor in some cancers, such as astrocytoma, hematomas, and breast cancer (54). According to many literature reports, overexpression of miR-101 targets many genes (such as STMN1, RAB5A, ATG4D, EZH2, EED, SOX9, COX-2, ATP5A1, ATP5B, and KLF6) and regulates their expression in GBM cells. In our previous study, we also verified that overexpression of miR-101 directly targeted genes involving DNMT3A, ELFN2, PRDM16, and CPEB1 and downregulated their expression. Furthermore, miR-101 indirectly targeted LMO3, CPEB1, ELFN2, and PRDM16 and regulated the expression of these genes by reversing their methylation status. Here, we found that LRRC4 was not the direct target gene of miR-101, but miR-101 was capable of reversing the LRRC4 hypermethylation status and expression in GBM cells. Hereby, we propose that miR-101 could be as a small molecule that participates in the recovery of LRRC4 expression in GBM cells and further affects CD4^+^CCR4^+^ T cells in the GBM microenvironment. Fortunately, we verified that miR-101 inhibited Ti-Treg cell expansion facilitated Ti-Teff cell expansion and CD4^+^CCR4^+^ T cell chemotaxis by epigenetically targeting LRRC4 in GBM cells. LRRC4 knockdown blocked the secretion of cytokines mediated by miR-101. Our previous study has shown that DNMT3A, EED, and EZH2 are direct targets of miR-101 in GBM cells, and histone methylation modification and DNA methyltransferase are critical factors that regulate gene promoter methylation (43–46, 54). H3K27 could repress the gene promoter to inhibit gene expression. In our study, we showed that miR-101 reversed LRRC4 hypermethylation by directly targeting EZH2, EED, and DNMT3A to decrease H3K27me3 enrichment of the LRRC4 core promoter. High expression of LRRC4 suggested a good prognosis in classical GBM patients, and a similar tendency was observed in neural and proneural GBM patients. Accordingly, as a small molecule that participates in the re-expression of LRRC4, miR-101 may serve as a potential therapeutic molecule for GBM, mainly for classical GBM treatment. In conclusion, this study demonstrated that LRRC4 re-expression in GBM cells mediated the accumulation of GBM-infiltrating CD4^+^CCR4^+^ T cells through cytokines and exosomes. In GBM cells, LRRC4 directly bound to PDPK1 and HSP90 and activated PDPK1/IKKβ/NF-κB signaling to promote cytokine secretion. miR-101 could be a small molecule capable of reversing LRRC4 expression through epigenetic mechanisms to mediate the interaction of GBM cells and GBM-infiltrating CD4^+^CCR4^+^ T cells (Figure 6). Our results identify a novel mechanism in the GBM microenvironment and suggest a new treatment method using LRRC4 re-expression in GBM cells to create a permissive intratumoral environment.

## Materials and Methods

### Collection of Human Biological Samples and Primary Tumor Cell Culture

Human clinical sample and data were collected from the Department of Neurosurgery, Central South University. All human experiments were performed in accordance with the Declaration of Helsinki and approved by the Joint Ethics Committee of the Central South University Health Authority. All subjects provided informed written consent. Primary tumor samples were minced with a GentleMACS Dissociator (Miltenyi Biotec). Cells were cultured in DMEM/F12 containing 10% FBS. Primary tumor cells were tested by GFAP, nestin, and CD133 staining and subcutaneous implantation in nude mice.

### Immunohistochemical Staining

The immunohistochemical experiment was performed using the Ultra Sensitive SP Kit (Maixin Biotechnology Company). Two independent pathologists who were blinded to the clinical pathological information performed the scoring.

### Flow Cytometry

Peripheral blood mononuclear cells and TILs were thawed in PBS and washed in fluorescence-activated cell sorting staining buffer. Single cell suspensions were assessed using a BD Biosciences LSRII flow cytometer and fluorochrome-conjugated antibodies against CD4, CD25, CD127, Foxp3, CD304 (neuropilin1), CCR4, CCR2, CD44, and CD62L. The results were analyzed using FlowJo software.

### Luciferase Reporter Assay

This procedure was carried out as previously described (55). Firefly and Renilla reniformis luciferase activities were measured 24 h after transfection using the dual-luciferase reporter assay system (Promega, Madison, WI, USA).

### Migration Assay

Tumor-infiltrating lymphocytes were seeded in the upper part of a Boyden chamber; the lower part contained tumor cell-derived exosomes (56, 57). The percentage of migrating cells was evaluated by flow cytometry (BD Biosciences LSRII flow cytometer), and the results were analyzed using FlowJo software.

### Co-Immunoprecipitation

For the LRRC4-PDPK1, LRRC4-HSP90, and PDPK1-HSP90 interactions, 10^7^ cells were prepared and lysed with GLB^+^ buffer. Antibodies were incubated with the cell lysate, and then the solution was incubated with Protein G beads (Thermo Scientific, 20399). After the beads were boiled, the lysates were subjected to Western blotting.

### GST Pull-down Assay

Bacterial lysates containing GST-PDPK1 domains or GST-HSP90 domains were incubated with glutathione-Sepharose 4B beads. The beads were incubated with cell lysates containing LRRC4, allowing the LRRC4-PDPK1 or LRRC4-HSP90 interaction. The interacted proteins were eluted and subjected to electro-phoresis.

### ChIP Assay

This procedure was carried out as previously described (54). A total of 2 × 10^7^ cells were used for the ChIP assay. Antibodies specific to trimethylated H3K27, dimethylated H3K4, trimethyl-H4K20 and trimethylated H3K9 were used (Millipore). Immunoprecipitated DNA from ChIP analyses with anti-H3K27me3, anti-H4K20me3, anti-H3K9me3, and anti-H3K4me2 antibodies was subjected to a RT-qPCR experiment.

### Statistical Analysis

All experiments were performed three times, and the data were analyzed with GraphPad Prism 5. Differences between the variables of the groups were tested using the Student’s *t*-test and Kaplan–Meier curves analysis, among others. A *P*-value < 0.05 was considered statistically significant.

## Ethics Statement

This study was carried out in accordance with the recommendations of “Joint Ethics Committee of the Central South University Health Authority” with written informed consent from all subjects. All subjects gave written informed consent in accordance with the Declaration of Helsinki. The protocol was approved by the “Joint Ethics Committee of the Central South University Health Authority.”

## Author Contributions

PL mainly performed the project. JF performed the vector construction. YL, CL, TL, and CZ helped with the experiments and analyzed the data. LF, WW, and GL helped approve the final version of the manuscript. QiangL, XS, and QingL prepared the clinical samples. MW developed the experimental design. All the authors approved the final manuscript.

## Conflict of Interest Statement

The authors declare that the research was conducted in the absence of any commercial or financial relationships that could be construed as a potential conflict of interest.
