# Supplementary material for: Novel Therapy for Glioblastoma Multiforme by Restoring LRRC4 in Tumor Cells: LRRC4 Inhibits Tumor-Infitrating Regulatory T Cells by Cytokine and Programmed Cell Death 1-Containing Exosomes
Source: Front Immunol. 2017 Dec 11;8:1748. doi: 10.3389/fimmu.2017.01748 (PMC5732324; doi:10.3389/fimmu.2017.01748)
Supplement: Supplementary file 1 [file Presentation_1.PPTX]

## Slide 1
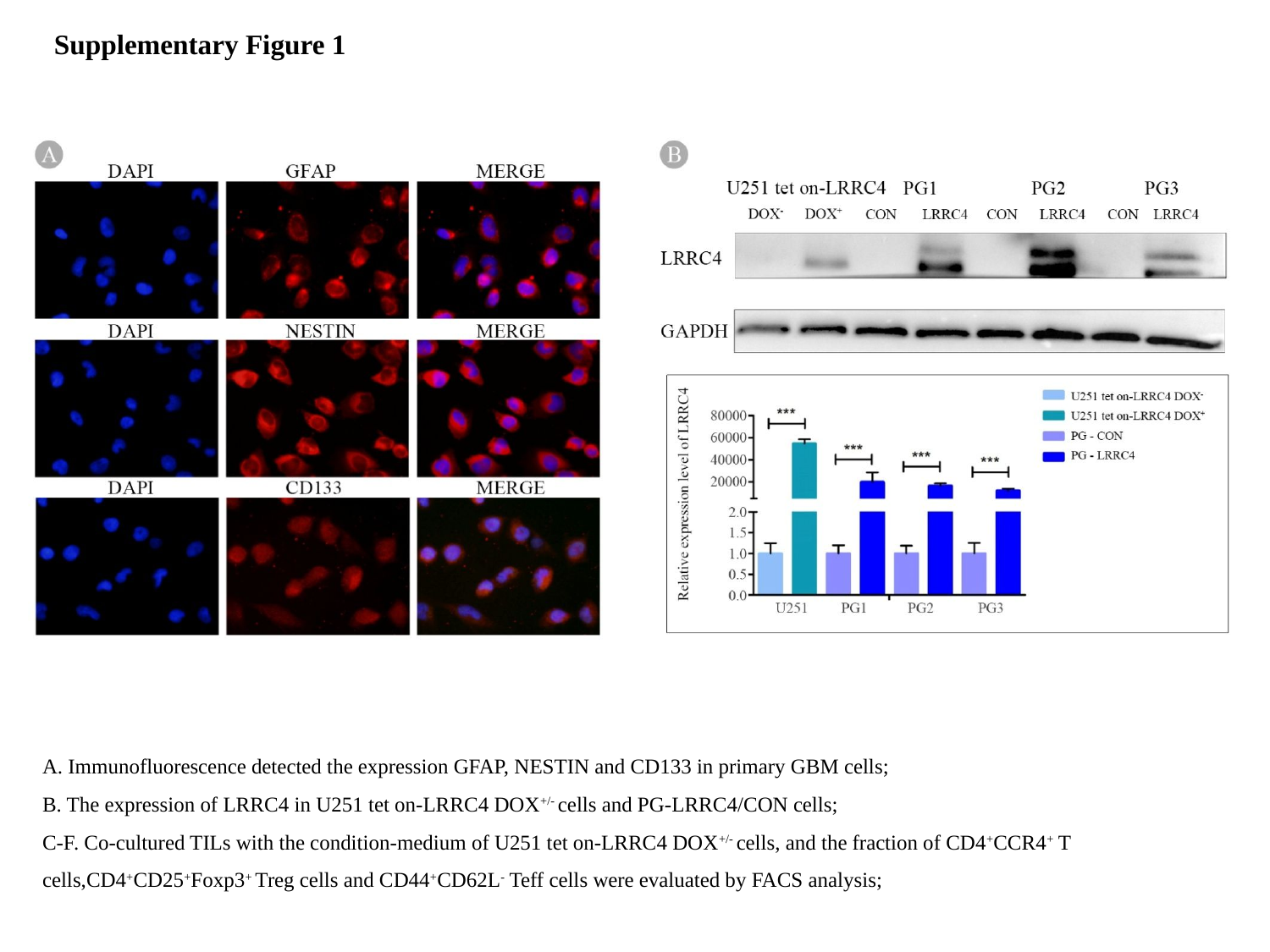

Supplementary Figure 1
A. Immunofluorescence detected the expression GFAP, NESTIN and CD133 in primary GBM cells;
B. The expression of LRRC4 in U251 tet on-LRRC4 DOX+/- cells and PG-LRRC4/CON cells;
C-F. Co-cultured TILs with the condition-medium of U251 tet on-LRRC4 DOX+/- cells, and the fraction of CD4+CCR4+ T cells,CD4+CD25+Foxp3+ Treg cells and CD44+CD62L- Teff cells were evaluated by FACS analysis;

## Slide 2
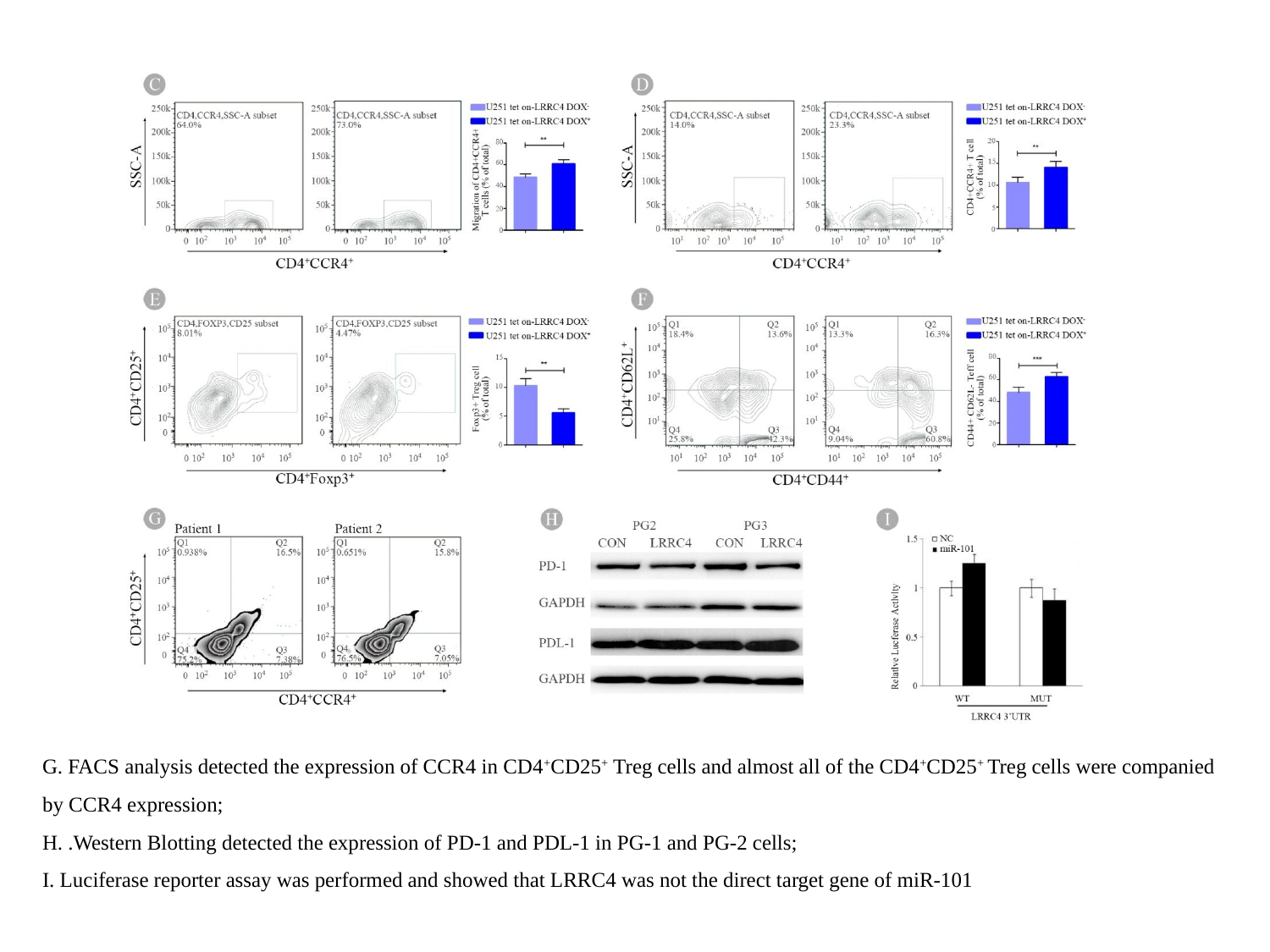

G. FACS analysis detected the expression of CCR4 in CD4+CD25+ Treg cells and almost all of the CD4+CD25+ Treg cells were companied by CCR4 expression;
H. .Western Blotting detected the expression of PD-1 and PDL-1 in PG-1 and PG-2 cells;
I. Luciferase reporter assay was performed and showed that LRRC4 was not the direct target gene of miR-101

## Slide 3
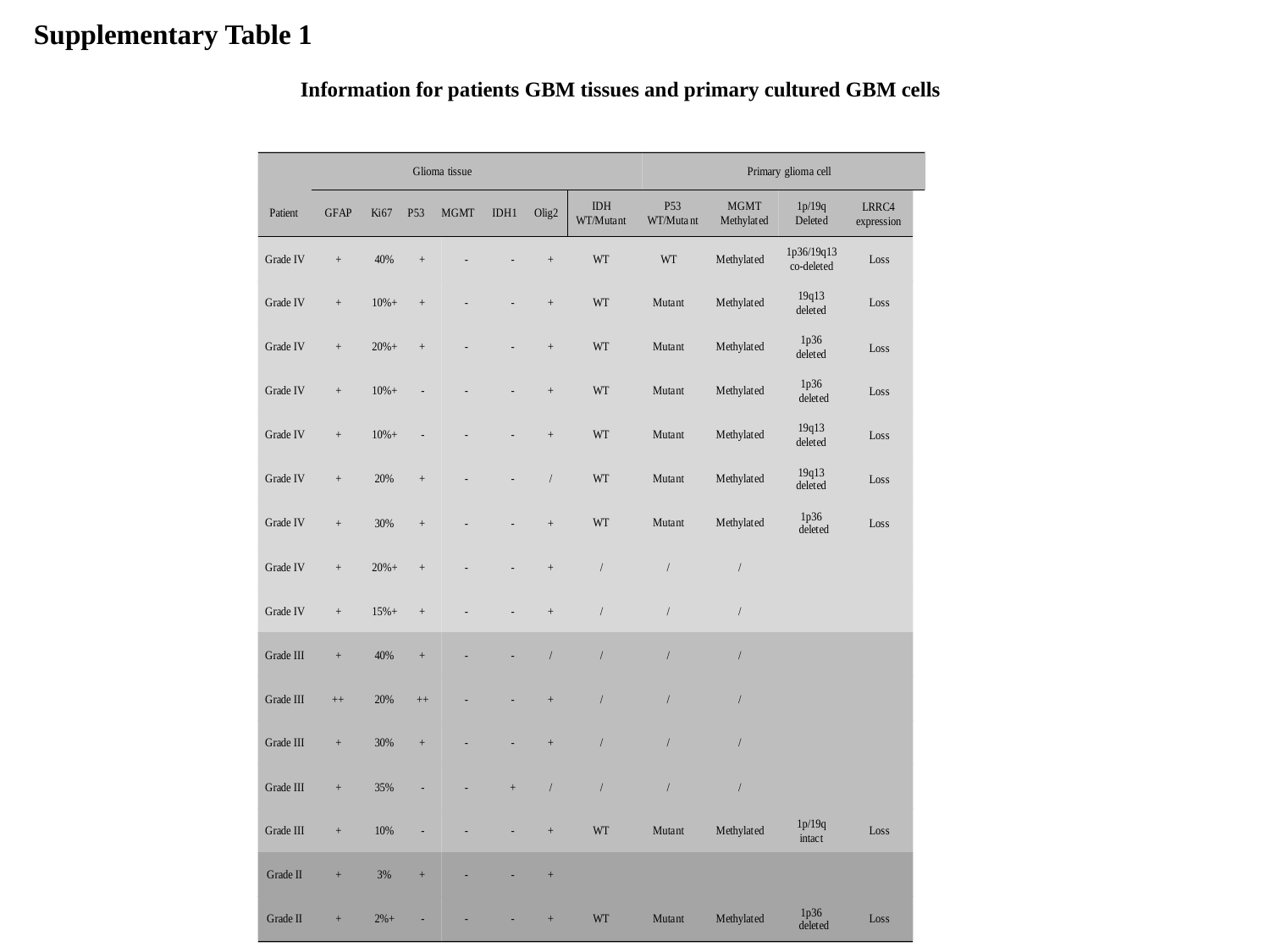

Supplementary Table 1
Information for patients GBM tissues and primary cultured GBM cells
